# Next-Generation Probiotics and Their Metabolites in COVID-19

**DOI:** 10.3390/microorganisms9050941

**Published:** 2021-04-27

**Authors:** Thomas Gautier, Sandrine David-Le Gall, Alaa Sweidan, Zohreh Tamanai-Shacoori, Anne Jolivet-Gougeon, Olivier Loréal, Latifa Bousarghin

**Affiliations:** 1 UMR 1241, Nutrition Metabolisms and Cancer Institute, Inserm, INRAE, Université de Rennes 1, 35000 Rennes, France; thomas.gautier@univ-rennes1.fr (T.G.); sandrine.legall-david@univ-rennes1.fr (S.D.-L.G.); zohreh.shacoori@univ-rennes1.fr (Z.T.-S.); anne.gougeon@univ-rennes1.fr (A.J.-G.); olivier.loreal@univ-rennes1.fr (O.L.); 2 Laboratory of Microbiology, Department of Life and Earth Sciences, Faculty of Sciences I, Lebanese University, Hadath Campus, P.O. Box 6573/14 Beirut, Lebanon; alaa.sweidan@hotmail.com

**Keywords:** SARS CoV-2, next-generation probiotic, butyrate, secondary bile acids, desaminotyrosine

## Abstract

Since December 2019, a global pandemic has been observed, caused by the emergence of a new coronavirus, SARS CoV-2. The latter is responsible for the respiratory disease, COVID-19. The infection is also characterized by renal, hepatic, and gastrointestinal dysfunctions suggesting the spread of the virus to other organs. A dysregulated immune response was also reported. To date, there is no measure to treat or prevent SARS CoV-2 infection. Additionally, as gut microbiota composition is altered in patients with COVID-19, alternative therapies using probiotics can be considered to fight SARS CoV-2 infection. This review aims at summarizing the current knowledge about next-generation probiotics (NGPs) and their benefits in viral respiratory tract infections and in COVID-19. We describe these bacteria, highlighted by studies using metagenomic approaches. In addition, these bacteria generate metabolites such as butyrate, desaminotyrosine, and secondary bile acid, suggested to prevent viral respiratory infections. Gut microbial metabolites transported via the circulation to the lungs could inhibit viral replication or improve the immune response against viruses. The use of probiotics and/or their metabolites may target either the virus itself and/or the immunologic process. However, this review showed that more studies are needed to determine the benefits of probiotics and metabolite products in COVID-19.

## 1. Introduction

Since December 2019, a global pandemic has been observed. It was caused by the emergence of a new coronavirus, the SARS CoV-2, characterized by a respiratory disease (COVID-19, coronavirus disease-19) which can lead to respiratory distress and be life-threatening [1]. The SARS CoV-2 virus is a *Coronaviridae* of the genus *Betacoronavirus*, also including SARS CoV (2003) and MERS CoV (2012). During a severe form of SARS CoV-2 infection, renal, hepatic, and gastrointestinal dysfunctions are observed. It is not yet clear whether they are related to the spread of the virus’s impact to other organs and/or to the systemic consequences of hypoxia and immune response. A dysregulated immune system was reported in a cohort of 452 patients with laboratory-confirmed COVID-19 [2]. An increase in the neutrophil to lymphocyte ratio (NLR), and T lymphopenia, including a decrease in CD4+ T cells, was common among patients with COVID-19 [2]. This indicates a serious disturbance in the immune environment that likely reflects a critical condition in severely infected cases. Similar to SARS and MERS, higher serum levels of pro-inflammatory cytokines (TNF-α, IL-1, and IL-6) and chemokines (IL-8) were found in patients with severe COVID-19 forms compared to individuals with mild disease. Models of animals with SARS CoV and MERS CoV showed marked inflammatory and immune responses which may activate a cytokine storm, leading to vascular leakage, and abnormal T-cell and macrophage responses. This will induce acute lung injury, acute respiratory distress syndrome, or even death [3]. Based on the genetic homology and pathologic features of the infected lung, a cytokine storm was predicted to prevail in patients severely affected during SARS CoV-2 infection [4]. It has been shown to have a marked increase in interleukin 1β (IL-1β), interferon γ (IFN-γ), interferon-inducible protein 10 (IP-10), and monocyte chemoattractant protein 1 (MCP-1).

Unfortunately, to date, there is no definitive medication to prevent or treat SARS CoV-2 infection. Several treatments are currently being evaluated in a strategy of repositioning [5]. Despite the fact that encouraging results are emerging, other complementary tracks must be followed to confirm their efficiency. This review aimed to summarize the current knowledge about next-generation probiotics (NGPs) and their metabolites to prevent virus-induced respiratory infectious diseases, such as COVID-19.

## 2. COVID-19 and Gut Microbiota

Several studies indicated that gut microbiota composition was altered in patients with COVID-19 [6,7,8]. They had intestinal microbial dysbiosis with decreased *Lactobacillus* and *Bifidobacterium* [9]. Indeed, other bacteria are also disrupted. *Collinsella*, *Streptococcus*, *Morganella* were more abundant in patients with high SARS CoV-2 infectivity, whereas *Parabacteroides*, *Bacteroides*, *Alistipes,* and *Lachnospiraceae* had higher abundances in patients with low to no SARS CoV-2 infectivity [10]. COVID-19 has been mainly fatal in elderly patients where gut microbiota diversity is decreased [11]. The most prevalent commensals in a healthy population with a median age of 48 years old are *Eubacterium*, *Faecalibacterium prausnitzii*, *Roseburia*, and *Lachnospiraceae taxa*, while in COVID-19 patients with a median age of 55 years old, these commensals decrease, and opportunistic pathogens, such as *Clostridium hathewayi*, *Actinomyces viscosus*, and *Bacteroides nordii*, are increased [12]. Several gut commensals with known immunomodulatory potential, such as *Faecalibacterium prausnitzii*, *Eubacterium rectale,* and bifidobacteria, were underrepresented in COVID-19 patients [13].

By using a non-human primate model (the macaque) that recapitulates mild COVID-19 symptoms, it was described that SARS CoV-2 infection is associated with changes in the gut microbiota’s composition and functional activity [14].

## 3. Probiotics as a Useful Treatment in Viral Respiratory Infections

Complementary and alternative therapies have been proposed worldwide to cure or prevent respiratory viral infections. Several viruses are known to cause respiratory infections in humans. Causative agents include respiratory syncytial virus (RSV), parainfluenza virus, enterovirus (EV), coronavirus, influenza virus, and adenovirus [15]. Probiotics have been proposed as a potential treatment during a viral respiratory infection. Food and Agriculture Organization (FAO) of the United Nations and World Health Organization (WHO) have defined them as microorganisms that can induce beneficial health effects. They are known to balance the interaction between host gut microbiota and the immune system and may promote resistance against pathogens [16]. Thus, they have been suggested to prevent and treat viral respiratory tract infections [17]. Probiotics have been described to reduce acute upper respiratory tract infections [18]. In addition, meta-analyses on the probiotic effect on children suggested a reduced risk of the upper respiratory tract [19,20].

The most common probiotic strains, *Lactobacillus* and *Bifidobacterium*, have been used in several studies [21]. Many studies have shown that the oral administration of different strains of *lactobacilli* protects against influenza virus [22,23]. *Bifidobacterium* has shown anti-influenza virus potential as a probiotic by modulating the gut immune system [24,25,26].

## 4. NGPs Include Promising Strains

**Figure 1:**
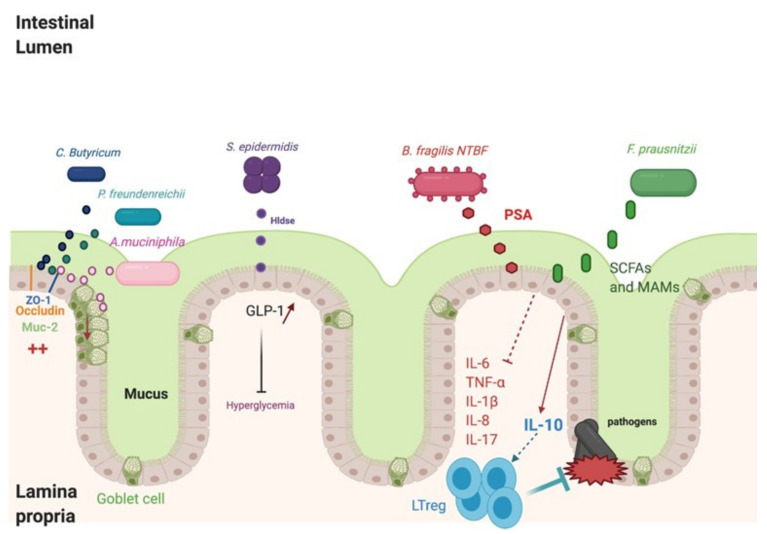
Impact of next-generation probiotics (NGPs) on intestinal epithelium. NGPs (non-toxinogenic *Bacteroides fragilis* (NTBF), *Faecalibacterium prausnitzii*, *Akkermancia muciniphila*, *Clostridium Butyricum*, *Propionobacterium freundenreichii*, *Staphylococcus epidermidis*) produce different metabolites impacting the health of the gut and other organs. The derived metabolites, polysaccharide A (PSA) of NTBF strain and short-chain fatty acids (SCFAs) and microbial ant-inflammatory molecules (MAMs) of *Faecalibacterium prausnitzii*, inhibit inflammatory cytokines and increase interleukin-10 (IL-10). *Clostridium Butyricum*, *Propionobacterium freundenreichii*, and *Akkermancia muciniphila* act by increasing zonula occludens-1 (Z0-1) and occludin expression improving the intestinal barrier. A. muciniphila restores the number of Goblet cells, reversing intestinal mucosa damage and increasing the expression of mucin-2 (MUC-2). *Staphylococcus epidermidis* Hldse can induce glucagon-like peptide-1 (GLP-1) expression in epithelial cells, decreasing hyperglycemia. Figure created with BioRender.com (accessed on 19 May 2020).

Traditional probiotics, such as *Bifidobacterium* and *lactobacillus*, are widely used. However, recent studies using metagenomic approaches have underlined the importance of commensal species in maintaining gut health and have helped to unravel many potential NGPs [27]. These include *Faecalibacterium prausnitzii*, *Akkermansia muciniphalis*, *Bacteroides fragilis*, and some strains of *Clostridium* (Figure 1). Recently, other bacteria, such as *Staphylococcus epidermidis*, *Enteroccocus hirae, Enterococcus faecium,* and *Propionibacterium freundenreichii*, have been shown to have beneficial effects [27,28,29].

### 4.1. Faecalibacterium prausnitzii

*Faecalibacterium prausnitzii* (*F. prausnitzii*) belongs to Firmicutes phylum, which is a Gram-positive bacterial group representing the majority of beneficial bacteria in the gut. Different in vitro studies have shown that it inhibits inflammation by decreasing inflammatory cytokines (tumor necrosis factor α (TNFα), and the interleukins, IL-1B, IL-6, and IL-8) and by increasing interleukin-10 (IL-10) [30]. Healthy effects were shown in mice by the inhibition of monocyte chemoattractant protein 1 (MCP1) and inducible nitric oxide synthase (iNOS) gene expression [31]. *F. prausnitzii* induced the dendritic cells to express a unique array of potent regulatory T-cell-polarizing molecules IL-10, IL-27, CD39, indoleamine-pyrrole 2,3-dioxygenase (IDO-1), and programmed death-ligand 1 (PDL-1), and inhibited their up-regulation of costimulatory molecules as well as their production of pro-inflammatory cytokines IL-12 and TNFα [30]. In association with Toll-like receptors (TLR) 2 and TLR6 heterodimer, *F. prausnitzii* also inhibits TLR4 expression, causing the gut inflammation to decrease [30].

Importantly, several studies have shown that *F. prausnitzii* administration could be used to treat disease in other organs, could improve hepatic health, and reduces adipose tissue inflammation in the context of high-fat-diet or high-fat diet-fed mice [32]. Derived from microbial anti-inflammatory molecules (MAMs), seven peptides were identified in the supernatant cultures of *F. Prausnitzii*. They have anti-inflammatory effects by inhibiting NF-ҡB in vitro, decreasing the levels of inflammatory cytokines IL-5, IL-8, and IL-17, and increasing the IL-10 level. *F. prausnitzii* is also known to produce other metabolites, such as short-chain fatty acids (SCFA), which have beneficial effects on health [33]. This is consistent with another study showing that children with asthma showed a decrease in the colonization of *F. prausnitzii* [34].

Recent data have shown that there is a relationship among the diet, immune system, and intestinal microbiome [35]. It is known that not only diet influences the composition of the intestinal microbiota, but also the microbiota and their products have an effect on the host [29]. These studies have highlighted that inulin can have a positive effect on the level of *F. prausnitzi* [36,37]. Xylo-oligosaccharide (XOS) supplementation was also determined to increase *Faecalibacterium sp*. and *Akkermansia sp*. as well as bifidobacteria but did not have a significant impact on the levels of lactobacilli [38,39].

### 4.2. Akkermansia muciniphila

*Verrucomicrobia* phylum contains *Akkermansia muciniphila*, which is a Gram-negative strain. These bacteria are known as NGPs [40]. *Akkermansia muciniphila* has an impact on immunity modulation, metabolic disorders, and epithelial cell protection in the gut [41]. *A. municiphila* helps to restore a healthy microbiota in the gut, decreases inflammatory cytokines (IL-6, TNF-α, and IL-12), and increases IL-10 cytokine [42]. *A. municiphila* administration increases the expression of tight junction proteins, zonula occludens-1 (Z0-1) and occludin in vitro and in vivo [43]. Another particularity of *A. muciniphila* is the induction of mucus production and Goblets cell proliferation in the intestinal crypt [44]. Mucin-2 (MUC-2), which is expressed in the small intestine and colon, forms a layer of mucus on the intestine to inhibit the adhesion and invasion of pathogenic bacteria [45]. *A. muciniphila* increased the expression of MUC-2 and Trefoil factor 2 (Tff2), which was also shown in a previous report. Tff2 are stable secretory proteins which aid in epithelium recovery via stabilizing the mucus layer [44]. For other mucins, such as MUC5AC and MUC5B, which are mainly expressed in the lungs, there is no study showing an impact of *A. muciniphila* on their expression [46]. Indeed, MUC5AC overexpression in mice decreased influenza virus (PR8 (Puerto Rico/8)/H1N1) infections and neutrophil responses [47]. The mechanism of host regulation is thought to involve derived materials, including pili-like proteins [48]. The outer membrane pili-like protein of *A. muciniphila,* Amuc_1100, has been identified to activate intracellular signals mediated by the TLR2 of intestinal epithelial cells, contributing to the enhancement of the intestinal barrier [49]. Amuc_1100 also appears to be involved in the immune response, in particular in the induction of the production of IL-10, an anti-inflammatory cytokine [49,50]. Additionally, the reduced abundance of *Akkermansia* in the intestinal tract has been linked with an enhanced risk of developing asthma [34].

However, A. *muciniphila* development is stimulated by polyphenols, which are considered as naturally existing substances in plants or obtained from foods (i.e., cereals, coffee, fruit, tea, vegetables, and wine). Derived from grapes, they act by increasing the abundance of *Akkermansia* in the intestinal tract; as a result, they have been shown to enhance intestinal barrier function and incretin secretion from intestinal endocrine cells. Cranberry administration has also been shown to enhance mucus secretion, which could create favorable conditions for *A. muciniphila*. The functionality of polyphenols has been deemed to contribute to the prevention of oxidative stress, which is a key solution for reducing the chronic pathologies associated with the establishment of dysbiosis. Hence, *A. muciniphila* and its Amuc_1100 proteins could be considered for the treatment of viral infection by inhibiting inflammation and stimulating mucus production.

### 4.3. Bacteroides fragilis

Bacteroidales are the most abundant families in the colon and are being studied as a potential NGP. One of them, the *Bacteroides fragilis* strain ZY-312, showed potentially health-promoting phenotypes when it was incubated with colonocytes and macrophages. *B. fragilis* is a Gram-negative anaerobic bacterium belonging to phylum Bacteroidetes that colonize naturally in the intestinal tract [51]. Some strains, such as Enterotoxigenic *B. fragilis* (ETBF), are toxic for the gut and cause diarrheal disease in children and adults [52]. The toxicity of these strains is caused by the presence of the pathogenicity island, BFPAI. The latter encodes *Bacteroides fragilis* toxin (BFT) responsible for diseases and colon cancer [53]. However, the major strains of *B. fragilis* are non-toxinogenic *B. Fragilis* (NTBF), which are considered beneficial for health. The *B. fragilis* ATCC 25,285 strain can induce Foxp3+ Treg activation, increase the level of IL-10 secretion, and decrease the IL-17 cytokine level in BALB/c mice [54]. The administration of a high level of ZY-312 to antibiotics-associated disease (AAD) rats caused an increase in MUC-2, ZO-1, and occludin expression. Some strains of NTBF, such as *B. fragilis* ATCC 25,285, secreted polysaccharide A (PSA) identified as an immunomodulatory molecule. PSA can interact with TLR2 on dendritic cells (DCs), which process PSA to interact with T-cell receptors. This interaction induces an expansion of IL-10-producing CD4+ cells [52]. PSA may provide robust protective anti-inflammatory responses during viral infections. In a study analyzing Herpes simplex infection in a murine model, it was observed that the PSA-treated mice exhibited high survival rates as compared to controls, and decreased levels of inflammation [55]. A comparison with other mice showed that IL-10 was the main anti-inflammatory factor secreted by CD4^+^ and CD8^+^ T cells. PSA can also inhibit asthma, and inflammatory bowel disease (IBD). Exposure to PSA resulted in the upregulation and secretion of IFNγ, TNFα, IL-6, and CXCL10, consistent with an interferon responsive gene (IRG) signature [56].

### 4.4. The Emergence of Other Bacteria

Recently, some studies have suggested the emergence of other bacteria less known as probiotics. It was shown that *Clostridium butyricum* induced a decrease in inflammatory cytokines (IFNϒ, TNF-α, IL-8, and IL-1β) and an increase in ZO-1 and occludin expression [57]. Hldse peptide of *S. epidermidis* may improve diabetes by glucagon-like peptide-1 (GLP-1) stimulation [58]. In addition, it was shown that *Propionibacterium freundenreichii* supernatant stimulated MUC-2 expression, decreased leucocyte infiltration and inflammation on Goblet cells, and decreased inflammatory cytokines (IL-6, TNF-α, and IL-1β) [59].

Respiratory commensal bacteria may be a source for developing NGP. The bacteria of the nasal human mucosa, *Corynebacterium pseudodiphtheriticum*, can be considered as a candidate to modulate respiratory immune responses and to fight against RSV [60]. In mice infected with RSV, *C. pseudodiphtheriticum* induces the secretion of IFN-β, TNF-α, IL-6, and IL-10, which may restrict inflammation during RSV infection and consequently reduces injury.

## 5. Probiotics Administration on Gut–Lung Axis in the Context of Respiratory Tract Infections

Different studies have described that the gut–lung axis is implicated in several diseases [61]. Some studies showed that lung diseases could be aggravated by gut microbiota disorder. Chronic obstructive pulmonary disease (COPD) is induced by nutrient deficiency associated with the decrease in anti-inflammatory molecules secreted by healthy bacteria [62]. Cystic fibrosis (CF) could also be aggravated by gut microbiota disorder in children. The decrease in *Bacteroides* spp. was associated with an increase in IL-8 and inflammation in cystic fibrosis patients [63]. Segmented filamentous bacteria in the gut enhance T_H_17/IL17A immune response and disrupt the immune homeostasis balance in the lungs. This leads to autoimmunity of rheumatoid arthritis (RA), which can generate lung complications in 59% of patients [64]. *Lachnospira* and *Clostridum neonatale* abundance is associated with asthma in children [65]. Additionally, a reduced abundance of *Akkermansia*, *Bifidobacteria*, and *Faecalibacteria* in the neonatal gut has been linked with a risk of developing asthma [34]. The intestinal microbiota not only modulate the balance of immunity in the gut but also in other organs.

Variations in the composition of the gut microbiota can lead to respiratory infections [61,66,67]. It was shown that avian H5N1 influenza A virus (IAV) decreased Bacteroidetes and increased Firmicutes seven days after infection. The depletion of the intestinal microbiota induced by IAV facilitates the invasion of bacterial pathogens [67]. Similarly, H7N9-infected patients showed a decreased microbial diversity in gut microbiota [68]. In human H7N9 infection, *F. prausnitzii*, *Bifidobacterium* spp., *Roseburia intestinalis*, and *lactobacillus* spp. were decreased, whereas pathogenic *E. coli* and *Clostridium butyricum* were increased [69]. RSV infection in mice also altered the microbiota composition: Bacteroidetes increased and Firmicutes decreased. Respiratory tract infections can impact the immune response in the lungs by modulating gut microbiota. Yu et al. (2015) suggested that modification in the gut microbiota may down-regulate the immune recognition mechanisms in the lungs. As a result, immune response functions could not be exerted, reducing the ability to clear viruses from the lungs [70].

Notably, gut–lung communication is bidirectional [61]. Inflammatory bowel disease (IBD) causes lung damage by increasing inflammation, leading to changes in tomography, and promotes respiratory tract infection [71]. Additionally, oral antibiotic treatment in mice worsens influenza A infection, showing the importance of the microbiota for respiratory tract infection [71]. Another study showed that cystic fibrosis is responsible for disrupting the gut microbiota. This supports the relationship between the lungs and the intestine. Dumas et al. (2018) concluded that the administration of probiotics (intranasal or oral) improves immunity and lowers pulmonary viral levels. This supports the relationship between the lungs and the intestine [72].

The oral administration of NGPs during viral pulmonary infection could help prevent intestinal infection and dysbiosis. It also helps fight viral pulmonary infections by reducing inflammation or acting directly on the virus with circulating bacterial metabolites. The latter produced by the gut bacteria move through the bloodstream to modulate the immune response in distant organs [73]. These metabolites play a key factor in the dialogue between the gut microbiota and the lungs. They can be derived from ingested nutrients as they cannot be found in food or produced by mammalian cells. They enter the portal vein reaching the liver where they will be metabolized. Molecules that were not metabolized enter the peripheral circulation to reach distal body organs.

## 6. Probiotic-Derived Metabolites as Beneficial Mediators in Viral Infection?

Many studies showed that gut microbiota contain healthy bacteria and the supernatant of these NGPs could help to fight against diseases in the gut and other organs [74,75]. Using NGPs could allow the discovery of new molecules to fight against respiratory virus infections. Several factors have been shown to be implicated alongside the gut–lung axis, including systemic dissemination of bacterial-derived components and metabolic degradation products.

**Figure 2:**
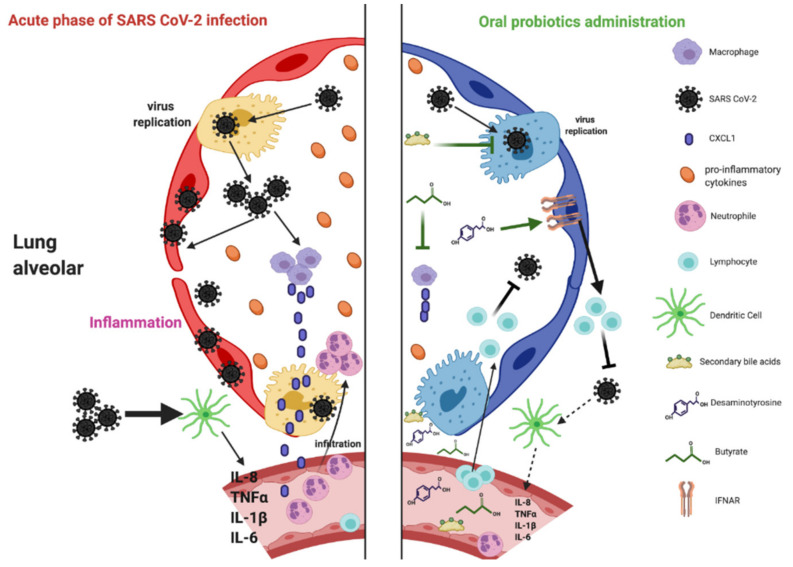
Model of the effect of next-generation probiotic (NGP)-derived metabolites on infected lungs. In the acute phase of SARS CoV-2 infection, inflammatory cytokines are secreted (storm cytokines), and airway debris and pulmonary edema, form leading to respiratory distress. After oral administration of NGPs, their metabolites are transported by the blood to the lungs where they can regulate the immune system or viral replication. Butyrate increases the proliferation of the macrophages that express a lower level of chemokine (C-X-C motif) ligand 1 (CXCL1), thus reducing neutrophil influx in the lungs. Secondary bile acid metabolites directly affect CD4^+^ T cells or decrease progeny viruses. Desaminotyrosine (DAT) metabolites boost type I interferons (IFN) production by amplifying interferon-α/β receptor (IFNAR) and activator of transcription 1 (STAT1) and activate lymphocytes. Figure created with BioRender.com (accessed on 19 May 2020).

Indeed, the intestinal microbiota generate metabolites that include folate, indoles, Gamma-aminobutyric acid, serotonin, secondary bile acids, desaminotyrosine (DAT), and also SCFAs (Table 1). We discuss here three of these metabolites (butyrate, DAT, and secondary bile acids) and their potential usefulness in treating respiratory tract viral infection as COVID-19 (Figure 2).

### 6.1. Butyrate

The most investigated bacterial metabolites are probably the SCFAs (ie, butyrate, propionate, and acetate). Several bacteria, *Lactobacillus* spp., *Bifidobacterium* spp., *Akkermansia muciniphila,* and *Bacteroides* spp., produce acetate [87], whereas propionate is produced by *Roseburia inulinivorans*, *Bacteroides* spp., *Veillonella* spp., and *Salmonella* spp. [88]. *Roseburia* spp., *F. prausnitzii,* and *Eubacterium rectale* generate butyrate. It was shown that the gut microbiota and their metabolites, including SCFAs, can regulate host inflammation and modulate the immunity against pathogens. Butyrate is considered an immunomodulatory compound. Additionally, it works as an essential energy source, allowing colonic cells to proliferate and maintain healthy gut barrier function [89]. Patients with butyrate-producing bacteria were less likely to develop viral lower respiratory infection (LRTI) [76]. A study on mice suggested that butyrate, by inhibiting histone deacetylase, restores IL-10 in the lung [90]. Furthermore, butyrate contributes to the healthy status of distant organs such as the lungs. Butyrate or propionate promotes the increase in lymphocyte antigen 6C (Ly6C) monocytes in the lungs during influenza infection [91]. These monocytes differentiate into activated macrophages, expressing a lower level of neutrophil chemoattractant (CXCL1). Thus, the influx of neutrophils is decreased, resulting in a reduced pulmonary immunopathology mediated by influenza.

### 6.2. Desaminotyrosine (DAT)

Another microbial metabolite called desaminotyrosine (DAT) joined SCFAs in affecting pulmonary response. It was shown that DAT protects mice against the influenza virus. This was also confirmed by Steed et al. (2017) who demonstrated DAT protection against influenza and that it decreases lung immunopathology [77]. Furthermore, the gut bacteria, *Clostridium orbiscindens*, were shown to produce DAT that rescued antibiotic-treated influenza-infected mice. DAT production occurs during flavonoid metabolism in specific species [92]. Flavonoids are a group of structurally diverse, water-soluble, polyphenolic, plant-derived compounds found in nutritionally diverse foods, including apples, berries, tea, citrus, and red wine [93]. These flavonoids activate type I IFN secretion and protect against influenza by increasing interferon-α/β receptor (IFNAR) and signal transducer and activator of transcription 1 (STAT1) signaling [94].

### 6.3. Secondary Bile Acids

In the liver, cholesterol is transformed into primary bile acids, cholic acid (CA), and chenodeoxycholic acid (CDCA). CDCA was shown to inhibit some viruses, such as rotavirus [95]. Some in vitro studies also revealed that CDCA inhibits three different influenza A virus (IAV) strains, including the pathogenic H5N1 [78]. Treatment with CDCA resulted in a decrease in viruses both in infected A549 and MDCK cells. CDCA can block viral ribonucleoprotein (vRNP) nuclear export, impairing IAV replication in vitro [78].

Gut bacteria can convert CA and CDCA into secondary bile acids, deoxycholic acid (DCA), lithocholic acid (LCA), and ursodeoxycholic acid (UDCA). *Bacteroides, Clostridium, Lactobacillus, Bifidobacterium, Clostridium,* and *Ruminococcus* are the main gut bacteria involved in bile acid metabolism. Some studies have begun to reveal the anti-inflammatory roles of bile acids, particularly in the innate immune system. They suppress NF-κB-dependent signaling pathways and inhibit NLRP3-dependent inflammasome activities [96]. Additionally, two CDCA-derived secondary bile acids, UCDA and hyodeoxycholic acid (HDCA), showed much weaker inhibitory activities against IAV. Additional anti-inflammatory roles for two distinct derivatives of LCA were revealed in both humans and rodents that directly affect CD4^+^ T cells: T helper cells expressing IL-17 (T_H_17) cell differentiation are inhibited by 3-oxoLCA, whereas regulatory T-cell (T_reg_) cell differentiation is enhanced by isoalloLCA [97].

## 7. Conclusions

At this time of the COVID-19 pandemic, when no pharmacological strategies for prevention or treatment are available, attention to alternative therapies using probiotics must be considered. Emerging data suggest that changes in the immune homeostasis, induced by SARS CoV-2, might be mediated by gut microbiota. Elderly people have less diverse intestinal flora in which beneficial microorganisms deteriorate [98]; this is in line with the observation that older individuals are more susceptible to SARS CoV-2 and more severe COVID-19 [99,100].

The structure and function of the intestinal flora could be a potential biological mechanism behind the diverse susceptibility of different groups of people to SARS CoV-2. The administration of these NGPs, isolated from the human gut, could restore the microbiota and improve the immunity in the lungs. Indeed, several studies have suggested a connection between the gut and the lungs. The interplay between these two organs may be based on soluble microbial metabolites transported via the circulation. The gut microbiota generate many diffusible metabolites that include secondary bile acids, DAT, and SCFAs.

NGPs can restore the immune response, either by correcting alterations of intestinal microbiota or by secreting metabolites that act in the lungs. Therefore, considering the potential beneficial impact of probiotics during respiratory viral infection, the possibility to use them as therapeutic agents during SARS CoV-2 infections should be considered. However, preclinical and clinical studies are needed to confirm the possible use of these NGPs or their metabolites in the fight against COVID-19.

## Figures and Tables

**Table 1:** Next-generation or common probiotic-derived metabolites implicated in diseases.

Metabolites	Main Bacteria Responsible for Production	Respiratory Tract Infection or Other Pathology	Mode of Action	Reference
Short-chain fatty acids (SCFAs) Butyrate	*Faecalibacterium*	Lower respiratory tract infection Influenza virus	Regulate inflammation, promote monocytes, decrease neutrophils, maintain gut barrier function.	[76]
Desaminotyrosine (DAT)	*Clostridium orbiscindens*	Influenza virus H1N1	Inhibit virus replication (interferons), decrease lung immunopathology.	[77]
Secondary bile acid	*Bacteroides*	Influenza virus H5N1	Inhibit virus replication, has anti-inflammatory properties.	[78]
Folate	*Lactobacillus*, *Bifidobacterium*	Gastric colorectal cancer	Involved in many metabolic pathways.	[79]
Indole derivatives	*Bacteroides thetaiotaomicron*	Metabolic syndrome, Psychiatric diseases	Affect host intestinal inflammation.	[80]
Gut-derived Neuro-transmitters (Serotonin, GABA, Histamine)	*L. reuteri* *Lactobacillus, Bifidobacterium* *L. reuteri*	Depression Anxiety and depression Allergy	Regulate numerous physiological processes, inhibit neurotransmitter (GABA), have several roles in immune functions.	[81] [82] [83]
Microbial Anti-Inflammatory molecules (MAMs)	*F. prausnitzii*	Colitis	Have anti-inflammatory properties	[84]
Amuc_1100	*A. muciniphila*	Obesity	Regulate host immunological homeostasis, and improve of gut barrier function	[85,86]

## Data Availability

Not applicable.
